# Plasticity of lifelong calorie‐restricted C57BL/6J mice in adapting to a medium‐fat diet intervention at old age

**DOI:** 10.1111/acel.12696

**Published:** 2017-12-21

**Authors:** Fenni Rusli, Mark V. Boekschoten, Vincenzo Borelli, Chen Sun, Carolien Lute, Aswin L. Menke, Joost van den Heuvel, Stefano Salvioli, Claudio Franceschi, Michael Müller, Wilma T. Steegenga

**Affiliations:** ^1^ Division of Human Nutrition, Nutrition, Metabolism & Genomics Group Wageningen University Wageningen The Netherlands; ^2^ Department of Experimental, Diagnostic and Specialty Medicine University of Bologna Bologna Italy; ^3^ TNO‐Triskelion Zeist The Netherlands; ^4^ Institute for Cell and Molecular Biosciences Newcastle University Newcastle Upon Tyne UK; ^5^ Laboratory of Genetics Wageningen University Wageningen The Netherlands; ^6^ Norwich Medical School University of East Anglia Norwich UK

**Keywords:** aging, DNA methylation, glycomics, liver, long‐term CR, NAFLD, transcriptomics

## Abstract

Calorie restriction (CR) is a dietary regimen that supports healthy aging. In this study, we investigated the systemic and liver‐specific responses caused by a diet switch to a medium‐fat (MF) diet in 24‐month‐old lifelong, CR‐exposed mice. This study aimed to increase the knowledge base on dietary alterations of gerontological relevance. Nine‐week‐old C57BL/6J mice were exposed either to a control, CR, or MF diet. At the age of 24 months, a subset of mice of the CR group was transferred to *ad libitum* MF feeding (CR‐MF). The mice were sacrificed at the age of 28 months, and then, biochemical and molecular analyses were performed. Our results showed that, despite the long‐term exposure to the CR regimen, mice in the CR‐MF group displayed hyperphagia, rapid weight gain, and hepatic steatosis. However, no hepatic fibrosis/injury or alteration in CR‐improved survival was observed in the diet switch group. The liver transcriptomic profile of CR‐MF mice largely shifted to a profile similar to the MF‐fed animals but leaving ~22% of the 1,578 differentially regulated genes between the CR and MF diet groups comparable with the expression of the lifelong CR group. Therefore, although the diet switch was performed at an old age, the CR‐MF‐exposed mice showed plasticity in coping with the challenge of a MF diet without developing severe liver pathologies.

## 1 INTRODUCTION

Aging has been described as an important risk factor for most chronic diseases, largely due to the impaired capacity to maintain homeostasis and resilience against environmental stress or damage at old age. For the liver, aging has been associated with an increasing risk to develop nonalcoholic fatty disease (NAFLD) (Argo, Northup, Al‐Osaimi, & Caldwell, 2009; Frith, Day, Henderson, Burt, & Newton, 2009). NAFLD covers a spectrum of liver diseases ranging from simple steatosis to nonalcoholic steatohepatitis (NASH), fibrosis, and cirrhosis. While hepatic steatosis is considered to be benign, NASH is the more severe condition that is characterized by inflammation and possibly, fibrosis. Aging has been linked to NAFLD development through a number of commonly shared molecular mechanisms associated with both the NAFLD/NASH development and hallmarks of aging, for example, reactive oxygen species formation, DNA damage, and hepatocyte senescence (López‐Otín, Blasco, Partridge, Serrano, & Kroemer, 2013). Aging and NAFLD are also intertwined with the modern obesogenic environment. The prevalence of obesity has been shown to increase at older age (Ford, Giles, & Dietz, 2002). Commonly observed consequences of obesity are elevated plasma insulin and free fatty acid levels. Due to these changes, free fatty acid uptake and triglyceride production in the liver increase, leading to the development of NAFLD (Karpe, Dickmann, & Frayn, 2011).

Calorie restriction (CR), a diet regimen of reduced energy intake without malnutrition, has been shown in numerous animal studies as by far the most effective approach to extend lifespan and to prevent age‐related and metabolic diseases (De Cabo, Carmona‐Gutierrez, Bernier, Hall Michael, & Madeo, 2014; Fontana & Partridge, 2015). Research on CR in humans has not been conclusive, but provides clues that beneficial metabolic adaptations observed in model species also occur in humans (Fontana, Partridge, & Longo, 2010; Mercken et al., 2013). The application of CR has been reported to be beneficial for liver health, by improving insulin sensitivity and reducing triglyceride accumulation in the liver (Eckard et al., 2013; Kirk et al., 2009).

To obtain health benefits of CR, a lifelong application of CR is required. In practice though, permanently reducing calorie intake is challenging for most individual, especially in an obesogenic environment prone to overfeeding. Only a very few individuals take up a CR regimen voluntarily, and even then, the feasibility of adhering to a CR diet is questionable. It has been shown in human intervention studies that adherence to CR decreases over time (Racette, 2006) and even a 5‐day‐a‐month CR regimen showed a dropout rate of 25% (Wei, 2017). This implies that people that have undertaken a CR regimen often return to an obesogenic diet. Studies concerning the response of long‐term calorie‐restricted subjects to *ad libitum* feeding are still limited, in particular the liver‐specific responses and NAFLD development at metabolic and transcriptomic level. A few mice studies were performed to model the long‐term CR situation. Giller and coworkers showed that the effects of 6‐month CR on adiposity, lipid profile in plasma and liver, and gene expression level completely diminish within 2 weeks of control diet feeding in C57BL/6JRj mice (Giller et al., 2013). Furthermore, a liver transcriptomic study by Dhahbi and colleagues reported that in 34‐month‐old B6C3F1 mice exposed to a CR diet for 21 months, 90% of the CR‐induced changes in gene expression in the liver disappear within 8 weeks of control diet feeding (Dhahbi, Kim, Mote, Beaver, & Spindler, 2004).

In this study, we investigate the consequences of 4‐month exposure to a Western diet after lifelong acquaintance to a CR diet. The CR regimen started at young age (2 months) and continued till old age (24 months), in order to model lifelong exposure to a CR diet. Between 24 and 28 months of age, we exposed a subset of mice in the CR group to an *ad libitum* medium‐fat (MF) diet, thereby generating a CR‐MF diet switch group. In the sacrificed 28‐month‐old mice, we investigated the systemic responses and NAFLD development following the diet switch at a physiological, metabolic, and molecular level. We explored both the plasticity of the mice at old age and the persistency of the effects caused by a lifelong CR diet.

## 2 RESULTS

### 2.1 Switching from a CR to MF diet at old age caused increasing body adiposity without affecting survival

**Figure 1:**
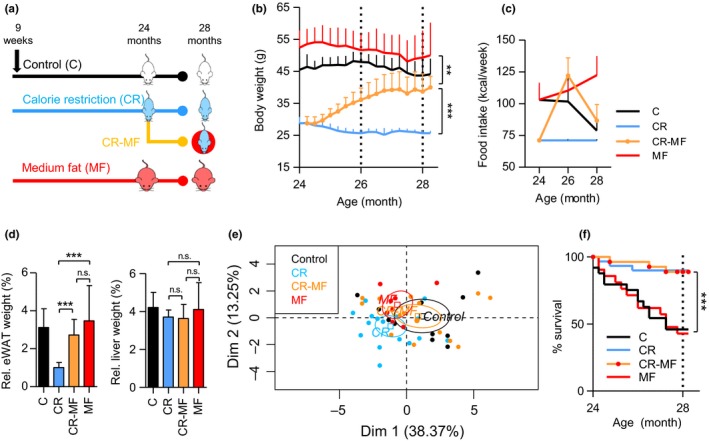
Physiological changes during the CR‐MF diet switch. (a) Experimental design. (b) Body weight development from 24 to 28 months of age following the diet switch. (c) Food intake measurement at 24, 26, and 28 months. (d) eWAT and liver weight. (e) Principle component analysis of 16 plasma inflammatory cytokines. (f) Kaplan–Meier survival curve, statistical difference was assessed by log‐rank analysis. *p <* *.05; **p <* *.01; ***p <* *.001

Nine‐week‐old C57BL/6J mice were randomly divided over three intervention groups and exposed to a control (C), calorie restriction (CR), or medium‐fat (MF) diet. At the age of 24 months, we replaced the diet of a subset of the CR intervention group by *ad libitum* exposure to the MF diet (CR‐MF diet switch) (Figure 1a). All mice were sacrificed at the age of 28 months. Figure 1b shows that, at the time point of the diet switch, the body weights of the C and MF‐exposed mice were substantially higher than those of the CR‐exposed mice. Following the transfer to the MF diet, the body weight of the CR mice dramatically increased (Figure 1b), reaching a new plateau at 27 months of age. At sacrifice, the body weight of the CR‐MF diet switch group was significantly higher than that of the lifelong CR‐fed mice, comparable to that of the C group, but still significantly lower than that of the MF‐exposed mice.

Food intake was recorded bimonthly and revealed that, transferring mice from the CR intervention group to *ad libitum* MF feeding, resulted in severe hyperphagia. Food intake of the CR‐MF group at the age of 26 months was even slightly higher than that of the lifelong MF intervention group (Figure 1c), although the difference was not significant (Fig. S1a). However, at the age of 28 months, when the body weight gain was stabilized at a new plateau, food intake of the CR‐MF mice decreased to similar amounts as consumed by the C intervention group.

Weight measurement of the epididymal white adipose tissue (eWAT) and liver revealed significant increases in the CR‐MF group compared with the lifelong CR‐exposed mice (Fig. S1b). However, after normalization to body weight, a significant increase was observed in the CR‐MF group only in relative eWAT weight, while no significant difference was found for the relative liver weight (Figure 1d).

As CR is known to protect against aging‐related low‐grade systemic inflammation, which is also called inflammaging (Franceschi et al., 2000), a panel of 16 inflammatory markers, including interferon gamma (IFNγ), tumor necrosis factor (TNF), interleukin‐1α (IL‐1α), IL‐1β, IL‐2, IL‐6, IL‐7, IL‐10, IL‐15, chemokine (C‐C motif) ligand 2 (CCL2 or MCP1), CCL3 (MIP‐1α), CCL4 (MIP‐1β), CCL5 (RANTES), chemokine (C‐X‐C motif) ligand 1 (CXCL1 or KC), CXCL9 (MIG), and CXCL10 (IP‐10), was measured in plasma to characterize the inflammatory status following the exposure to MF diet. The principal component analysis (PCA) plot presented in Figure 1e revealed that the plasma inflammatory profile of the CR‐MF switched animals had shifted into the direction of the C‐fed mice.

The survival rate recorded between 24 and 28 months revealed that mortality of the CR‐MF diet switch group was equivalent to the lifelong CR‐exposed mice and strongly different from the C and MF intervention groups (Figure 1f). Therefore, despite the changes in whole‐body adiposity, the improved survival gained in the CR period was successfully maintained during the 4‐month exposure to the MF diet.

### 2.2 The liver transcriptome of the CR‐MF diet switch mice strongly shifted to the MF profile

**Figure 2:**
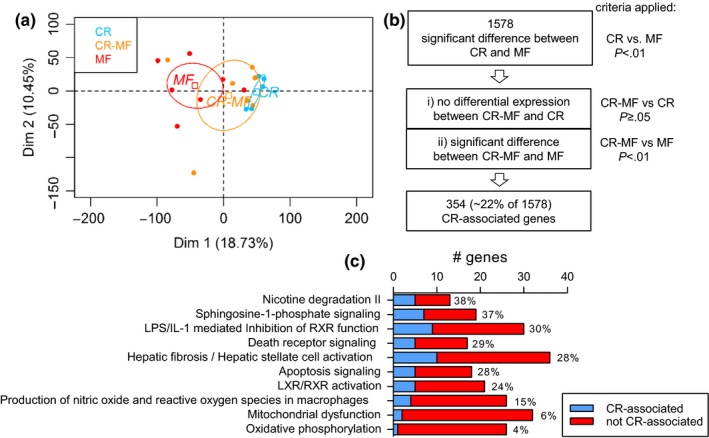
Liver transcriptomic profile of the CR‐MF diet switch largely altered toward the direction of the lifelong MF diet group. (a) Principle component analysis plot for individual animals showing the CR‐MF animals shifted to the cluster of MF diet group. The expression values of all three dietary interventions were normalized to the C group. (b) Analysis scheme for investigating the status of the CR‐differentially expressed genes after CR‐MF diet switch. A smaller proportion of differentially expressed genes remained similar to the expression of the lifelong CR, while most of the genes shifted toward the profile of MF's. (c) The fraction of the CR‐associated and not CR‐associated genes in the top 10 differentially regulated pathways between the lifelong CR and MF diet groups

To investigate the diet switch effect on the liver transcriptome of the CR‐MF diet group, a microarray analysis was performed. A PCA was carried out, using the C diet group as the reference. The results presented in Figure 2a show a remarkable shift in the transcriptome profile of the CR‐MF diet switch group from the CR toward the MF‐exposed mice. Notably, the interindividual variability in the MF diet group and in particular the CR‐MF group was much higher than in the CR group.

Although a pronounced switch in the transcriptomic profile of the CR‐MF‐exposed animals from the lifelong CR group was detected, the diet switch did not result in a complete overlap with the expression profile of the MF‐exposed group. To investigate which genes in the CR‐MF expression profile remained similarly expressed with that of the lifelong CR‐exposed mice, we applied the following gene screening (Figures 2b and S2a). We first identified the differentially expressed genes between the lifelong CR and MF groups (*p *<* *.01), which resulted in a list of 1,578 genes. Then, to determine which of these 1,578 genes remained comparable to the CR‐exposed mice after the CR‐MF switch and significantly different to those of the MF diet group, we screened for the genes (i) displaying no differential expression between the CR‐MF and CR groups (*p *≥* *.05) and (ii) displaying significant (*p *<* *.01) difference in expression between the CR‐MF and MF intervention groups. The direction of the fold change was also checked to confirm that the CR‐MF and CR groups show the same direction of change in comparison to the MF group. A subset of 354 “CR‐associated genes” were identified, exhibiting similar expression in the CR and CR‐MF groups. Figure S2b visualizes the expression levels of the 354 CR‐associated genes in the individual CR‐, CR‐MF‐ and MF‐exposed mice. Overall, we showed that, following the CR‐MF diet switch, 354 genes remained similarly expressed with the lifelong CR‐exposed mice (~22% of the 1,578 genes), while the majority (~78% of the 1,578 genes) adapted to the MF expression profile.

### 2.3 Functional characterization of the CR‐associated genes in the liver

Ingenuity pathway analysis (IPA) was applied to explore which canonical pathways were represented by the 1,578 genes displaying differentially expression between the CR and MF intervention groups. The 10 most significantly different canonical pathways listed in Table S1 revealed highly significant regulation of various pathways commonly acknowledged to be affected by CR, including mitochondrial dysfunction, oxidative phosphorylation, and apoptosis signaling. Next, we assigned the gene members in each pathway as a CR‐associated or MF‐adapted gene, according to the above‐explained criteria. The results presented in Figure 2c show that the pathways containing a relative high percentage of CR‐associated genes were found to be related to disease progression, for example, sphingosine‐1‐phosphate signaling, apoptosis, hepatic fibrosis/hepatic stellate cell activation. Meanwhile, oxidative phosphorylation and mitochondrial dysfunction, both of which are pathways related to energy utilization, only contained a small fraction of the CR‐associated genes (4% and 6%, respectively). This suggests that, following the CR‐MF diet switch, pathways related to energy utilization largely adapted to the expression profile of the MF diet group. In addition, these results suggest that the CR‐associated genes in the CR‐MF diet switch group are not confined to a specific pathway. We extended the analysis to CR‐regulated key metabolic pathways, which are known to be energy/nutrient sensing‐dependent (Anderson & Weindruch, 2010). PCA performed on AMPK, PI3K/AKT and insulin‐IGF (Fig. S3) signaling gene sets revealed that the expression profile of the CR‐MF diet switch mice again strongly shifted from the CR profile toward the lifelong MF cluster.

### 2.4 Identification of upstream regulators of the CR‐associated genes in the liver

Next, by applying IPA, we searched for predicted upstream regulators of the 354 CR‐associated genes. This analysis corroborates expression changes from multiple genes, which decreases the likelihood that any one that might be rendered insignificant by post‐transcriptional regulation would lead to a misleading biochemical/physiological surmise. The results presented in Table 1 show the top 10 predicted upstream regulators revealing highly significant *p*‐values (10^−10^–10^−3^). We observed that the regulators related to hepatic fibrosis including transforming growth factor β1 (TGFβ1), interleukin‐1β (IL‐1β), and hypoxia‐inducible factor 1‐α (HIF1α) were inhibited (activation *z*‐score <−2.000). Two of these regulators, IL‐1β and HIF1α, are also related to inflammation. Furthermore, acyl‐CoA oxidase 1 (ACOX1), which has been previously linked to NAFLD for its role in lipid metabolism, was predicted to be activated. ACOX1 activity represents fat oxidation, which helps to prevent the accumulation of fat in the liver.

**Table 1:** Upstream regulators of the 354 CR‐associated genes

Top 10 upstream regulator	Target genes (*n*)	*p*‐value^a^	Activation *z*‐score^b^	Predicted activation state^b^
ACOX1	17	1.32 × 10^−10^	3.153	Activated
TGFβ1	59	1.40 × 10^−9^	−3.581	Inhibited
AHR	19	1.04 × 10^−6^	2.523	Activated
IL‐1β	32	3.82 × 10^−6^	−2.774	Inhibited
COMMD1	5	1.56 × 10^−5^	2.236	Activated
Alpha‐catenin	10	1.94 × 10^−5^	2.618	Activated
BTNL2	8	2.61 × 10^−5^	−2.121	Inhibited
HIF1α	16	2.04 × 10^−4^	−2.140	Inhibited
ERK	11	9.71 × 10^−4^	−2.121	Inhibited
CD44	9	1.06 × 10^−3^	−2.449	Inhibited

a Based on previous knowledge of expected effects between upstream regulators and their target genes in the IPA database, upstream regulator analysis was performed. Top 10 predicted upstream regulators are presented.

b Another standard statistical measure in upstream regulator analysis in IPA is activation *z*‐score. The known effect (activation or inhibition) of an upstream regulator was compared with observed changes in gene expression. Based on the concordance between the two, an activation *z*‐score was determined, showing whether the predicted upstream regulator was activated (*z*‐score >2), inhibited (*z*‐score <−2) or uncertain.

### 2.5 Development of hepatic steatosis, but no hepatic fibrosis/injury, in the CR‐MF diet switch group

**Figure 3:**
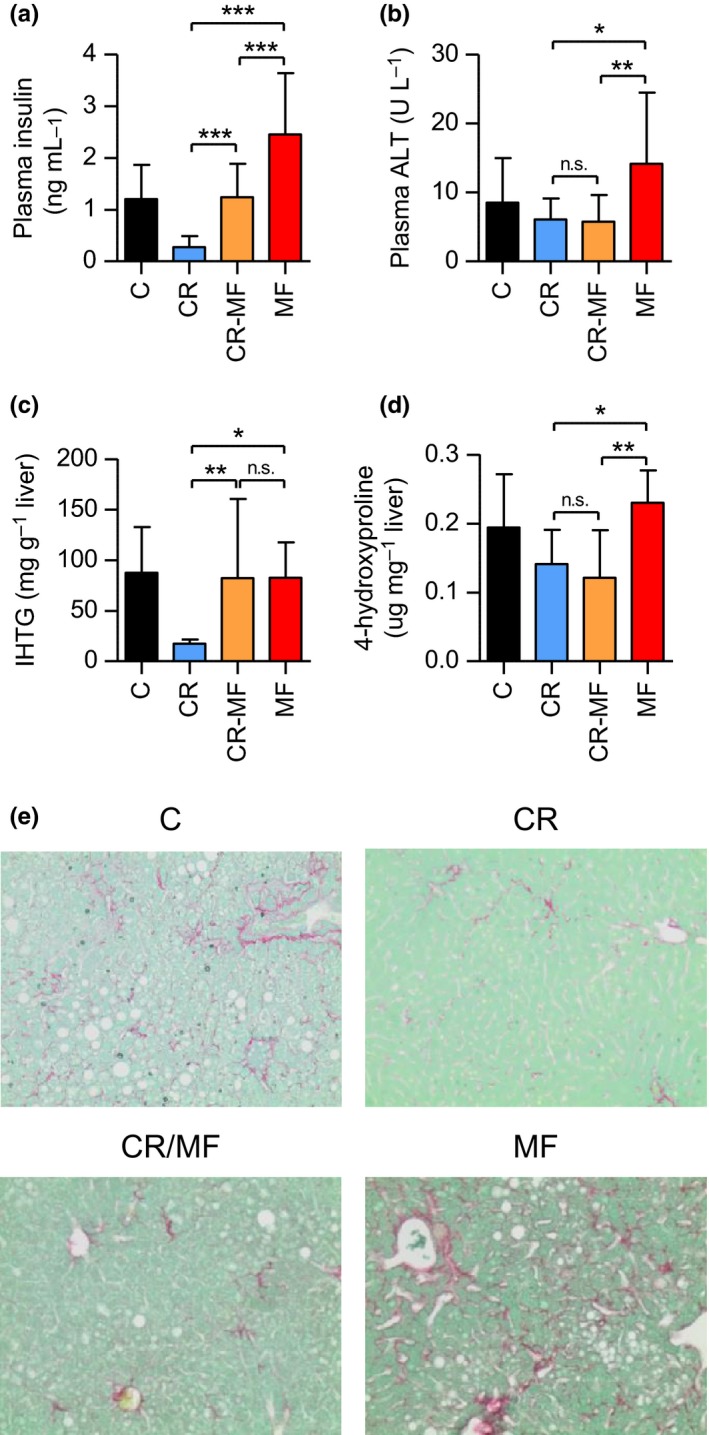
The CR‐MF diet switch group demonstrated elevated insulin and IHTG levels, but comparable plasma ALT and liver hydroxyproline compared to the CR group. (a) Fasting plasma insulin level. (b) Plasma ALT. (c) IHTG content. (d) Liver 4‐hydroxyproline content. Statistical difference was determined by one‐way ANOVA followed by Tukey post‐test. *p <* *.05; **p <* *.01; ***p <* *.001. (e) Collagen staining on liver sections (original magnification 200×)

Next, we explored the CR‐MF diet switch effect on NAFLD development in more detail. Firstly, the measurement of plasma insulin level, one of important factors in the pathogenesis of NAFLD, revealed that the level increased in the CR‐MF mice in response to the diet switch, but did not reach the level of the lifelong MF‐exposed animals (Figure 3a). Plasma glucose levels of the animals in CR‐MF diet switch did not significantly differ from other groups (Fig. S4a). The plasma alanine transaminase (ALT) level, a marker for liver injury, was markedly elevated only in the lifelong MF diet group but not in any of the other intervention groups (Figure 3b). The measurement of hepatic steatosis, which is represented by intrahepatic triglyceride (IHTG) content, revealed that after the diet switch the level of fat accumulation in the liver of the CR‐MF diet group significantly increased to a level comparable to the lifelong MF‐exposed mice (Figure 3c). The measurement of liver 4‐hydroxyproline content, a marker for hepatic fibrosis, showed elevated levels in the lifelong MF‐exposed animals, but not in the CR‐MF group (Figure 3d). This result was confirmed by collagen staining of liver sections (Figures 3e and S4b). To summarize, the 4 months exposure to MF diet resulted in elevated insulin and IHTG levels, but did not induce the progression of NALFD to liver fibrosis and injury.

### 2.6 CR‐MF diet switch shifted plasma N‐glycomics profile without altering the gene expression level of *Fut8*, one of the major glycosyltransferase

**Figure 4:**
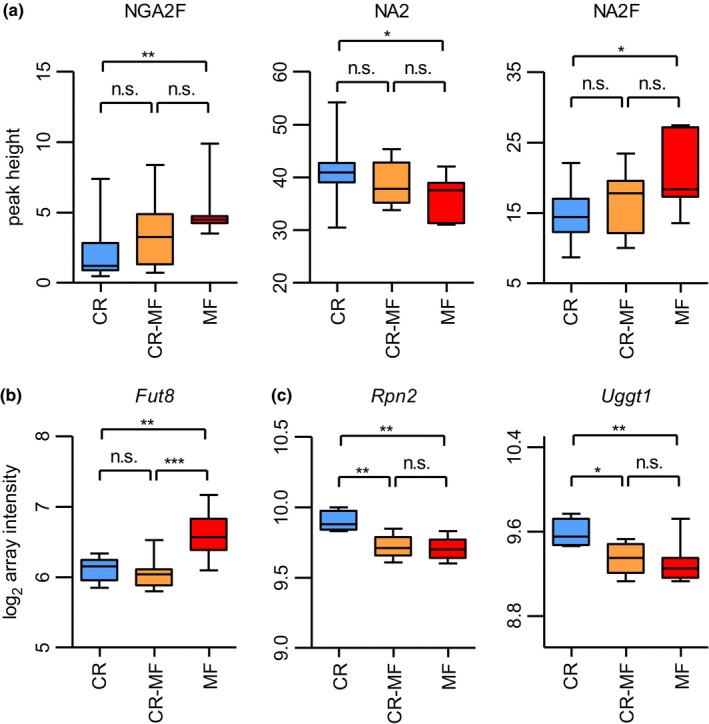
Plasma N‐glycosylation profile and expression levels of glycosylation modifying genes *Fut8*,* Rpn2,* and *Uggt1*. (a) Plasma levels of three N‐glycans, NGA2F (peak 1), NA2 (peak 5), and NA2F (peak 6) had shifted following the CR‐MF diet switch. Statistical significance was assessed by one‐way ANOVA followed by Tukey post‐test analysis. (b) The expression levels of CR‐associated gene *Fut8* in the CR‐MF diet switch group remained similar to those of CR and significantly differed from the expression levels of MF's. (c) Following the diet switch, gene expression levels of *Rpn2* and *Uggt1* in the CR‐MF group decreased. Statistical difference for the gene expression data was determined by intensity‐based moderated *t*‐statistic (IBMT) *p*‐value. *p <* *.05; **p <* *.01; ***p <* *.001

Previous studies have shown that plasma N‐glycosylation profiles are associated with chronic liver diseases (Blomme et al., 2011). The results presented in Figure 4a show that all three major N‐glycan structures previously identified (Vanhooren et al., 2011), bigalactosylated, biantennary glycan (NA2), agalactosylated, core‐α‐1,6‐fucosylated biantennary glycan (NGA2F) and bigalactosylated, core‐α‐1,6‐fucosylated biantennary glycan (NA2F), were significantly different between the lifelong CR and MF diet groups. The levels of NA2, NGA2F, and NA2F in the CR‐MF diet group were in between those of the CR‐ and MF‐fed animals, but did not differ significantly from either of the two intervention groups. Previous research has shown that expression and activity of α‐1,6‐fucosyltransferase (*Fut8)* in the liver were strongly associated with the plasma profiles of the three N‐glycan structures (Vanhooren et al., 2011). In our study, *Fut8* expression differed significantly between CR and MF‐exposed mice (Figure 4b; confirmed by qPCR analysis in Fig. S5). However, *Fut8* was one of the 354 CR‐associated genes of which the expression did not alter in response to the diet switch. We further explored other genes involved in glycosylation biosynthesis and degradation (Table S2) and found that a number of genes in these processes were strongly correlated with the three N‐glycan structures. Notably, NGA2F was correlated with ribophorin I (*Rpn1*), NA2 with mannosyl‐oligosaccharide glucosidase (*Mogs*) and hexosaminidase B (*Hexb*), and NA2F with ribophorin II (*Rpn2*) and UDP‐glucose glycoprotein glucosyltransferase 1 (*Uggt1*). *Rpn1* and *Rpn2* are genes in transferring glycans to asparagine residues, *Mogs* supports the trimming of N‐glycan in endoplasmic reticulum (ER), *Hexb* is involved in N‐glycans degradation, and *Uggt1* contributes to the regulation of N‐glycan quality control (Clarke, Novak, Lake, Hardwick, & Cherrington, 2017). Interestingly, expression levels of *Rpn2* and *Uggt1* differed significantly between CR and MF‐exposed mice, and the CR‐MF diet group showed similar expression levels to those of the MF group (Figure 4c). Therefore, this indicates that although *Fut8* did not change following the CR to MF diet switch, the altered glycosylation was influenced by other mechanisms involved in N‐glycan biosynthesis.

### 2.7 The relationship between physiological and gene expression level in hepatic steatosis development following the CR‐MF diet switch

**Figure 5:**
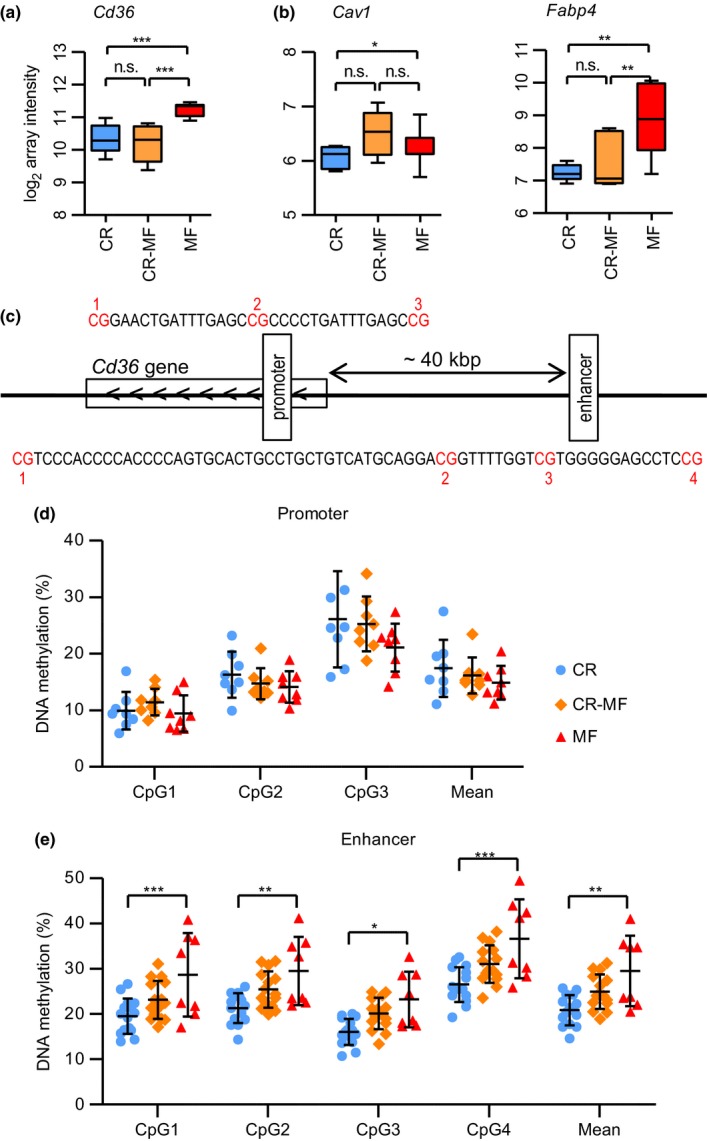
The gene expression levels of fatty acid uptake‐related genes *Cd36, Cav1,* and *Fabp4* and DNA methylation levels of *Cd36*. (a) The gene expression levels of CR‐associated gene *Cd36*. (b) The gene expression levels of *Cav1* and *Fabp4*. Statistical significance of the gene expression data was determined by intensity‐based moderated *t*‐statistic (IBMT) *p*‐value. (c) The location of promoter and enhancer region upstream of *Cd36*. (d) DNA methylation levels of CR, CR‐MF, and MF in the promoter region. (e) DNA methylation levels in the intergenic enhancer region. Differences on methylation levels were analyzed using two‐way ANOVA followed by post hoc Bonferroni test. *p <* *.05; **p <* *.01; ***p <* *.001

While IHTG levels were significantly increased in the CR‐MF diet switch group, cluster of differentiation 36 (*Cd36*), a key fatty acid transporter in the development of hepatic steatosis (Sheedfar et al., 2014), did not follow the same pattern. As one of the 354 CR‐associated genes which expression did not alter in response to the diet switch, *Cd36* displayed significant difference between the CR and MF intervention groups but its expression did not adapt to the MF diet after the diet switch (Figure 5a; confirmed by qPCR analysis in Fig. S5). Other fatty acid uptake/transporter genes were explored (Fig. S6 for genes without significant alteration), and we found that the expression levels of caveolin 1 (*Cav1*) (Figure 5b; confirmed by qPCR analysis in Fig. S4) and fatty acid binding protein 4 (*Fabp4*) (Figure 5b) were elevated in the CR‐MF diet group, implying that, while the *Cd36* expression was repressed, there were alternatives for fatty acid uptake.

### 2.8 Differentially methylated enhancer region was found in the intergenic region adjacent to *Cd36*

As *Cd36* plays an important role in the development of NAFLD, the mechanism behind its gene repression in the CR‐MF diet switch is of biological interest. A possible mechanism underlying the *Cd36* repression is by altering the DNA methylation of regions of the gene involved in transcription regulation. To explore this possibility, we analyzed the DNA methylation level of the promoter and enhancer region of *Cd36*. Promoter and weak enhancer regions were obtained from the mouse ChromHMM track (Ernst & Kellis, 2012) and identified to be present in the gene body and a distant upstream region, respectively (Figures 5c and S7 for detailed chromosomal position and epigenetic features). As shown in Figure 5d, we did not find a significant difference for the methylation level in the promoter region. However, the methylation levels of all 4 CpG sites analyzed in the enhancer region were significantly higher in the MF diet group compared to the levels in CR group (Figure 5e). Interestingly, although the methylation levels of the CR‐MF diet switch group were slightly increased compared to the lifelong CR mice, the methylation percentage of each of the CpGs was markedly lower compared to the lifelong MF‐exposed animals.

## 3 DISCUSSION

In this study, we aimed to investigate the systemic and liver‐specific responses of 24‐month‐old, lifelong CR‐exposed mice to 4 months of MF intervention. Our data revealed that in the diet switch group, most of the CR‐related features shifted to the MF profile: (i) whole‐body adiposity, (ii) hepatic steatosis, (iii) global transcriptome, and (iv) CR‐specific molecular features including IGF‐1/insulin signaling, oxidative phosphorylation, and AMPK signaling. These results show that the CR‐MF‐exposed animals have great plasticity in coping with the challenge of the MF diet. We also show that a number of CR‐related features were maintained in the CR‐MF group: (i) the prevention of hepatic fibrosis and injury, (ii) the improved survival, and (iii) the expression levels of a subset of CR‐related genes that were not altered by 4‐month exposure to the MF diet. Expanding the late‐life MF diet exposure period or challenging the mice with a high‐fat diet or acute liver injury would be of interest to further explore the protective effect induced by the CR diet.

Even after a long‐term exposure to the CR diet until an old age of 24 months, the mice were not adapted to the low energy intake and displayed extreme hyperphagia. The lifelong CR‐exposed mice in our aging cohort demonstrated their anticipation to receiving their daily food allotment by a burst in their activity level just prior to the regularly scheduled feeding (Van Norren et al., 2015). The hyperphagic response is an indicator that hunger persists even after a long‐term CR (Hambly, Mercer, & Speakman, 2007) and is maintained until body weight reaches the level of the *ad libitum*‐fed animals (Selman & Hempenstall, 2012). It is followed by a dramatic weight gain, adipose tissue expansion, and hepatic steatosis. This depicts a thrifty “catch‐up fat” characteristic, in which metabolic processes have evolved to be efficient in storing excessive energy once an energy supply is available (Barnes & Ozanne, 2011). Therefore, our experiment indicates that a lifelong application of the CR regimen is not able to acclimatize the mice to low energy intake and to resist the thriftiness, when the food availability is no longer restricted. The fact that the mice, even after lifelong exposure to a CR diet, are still not adjusted to the low‐calorie intake is something to take into consideration with regard to the human situation. If a constant hunger experience is the side effect of a CR diet, even at the long run, it can be questioned whether this suffering is compensated for by living a long and healthy life. Our results endorse the search for alternatives to CR regimen, in which food restriction is not continuously applied, for example, intermittent CR (Brandhorst et al., 2015; Rusli et al., 2015, 2017); or CR‐mimetics (Gillespie, Pickering, & Eskiw, 2016; Ingram & Roth, 2015), compounds that produces CR‐like effects on longevity without requiring the reduced food intake.

Food intake measurements revealed an unanticipated increase in MF diet group between 26 and 28 months of age, which was contrary to the typically decreasing intake at old age (Hamrick et al., 2006). In a study that compares young and old mice, it was revealed that the old mice drop significantly more food than the young ones, which possibly contributed to age‐related decline in oral motor function and/or dentition and/or joint impairment (Starr & Saito, 2012). Therefore, the correction for small pieces of food pellets spoiled in the cages could be crucial to avoid an overestimation of the food intake at old age.

Following the CR‐MF diet switch, the beneficial effects of a CR diet on the liver transcriptomic profile changed dramatically, confirming the results of previous investigations (Dhahbi et al., 2004; Giller et al., 2013). This could be explained by the dependence of a number of CR‐mediated pathways (e.g., oxidative phosphorylation, AMPK signaling) on energy depletion/stress (Burkewitz, Zhang, & Mair William, 2014). Despite the pronounced alteration of the transcriptomic profile, in our study, we found a set of 354 CR‐associated genes, which expression remained to be comparable with the expression levels of the lifelong CR intervention group. Possible explanations for this difference include the experimental settings, that is, mouse strains, the varying length of exposure to CR, severity of calorie reduction, type of diets used, age of mice at time point of observation, the use of different microarray platforms with different amounts of annotated genes (B6C3F1 mice, 44E% CR from 7 till 32 months, micronutrients supplementation to reach adequate level was not specified, fed at the beginning of light phase, followed by 8 weeks of control AIN‐93M diet, Affymetrix Mu11K sets A and B oligonucleotide arrays in Dhahbi et al. (2004); C57BL/6JRj mice, 25E% CR with micronutrients supplementation from 6 to 8 weeks of age for 6 months, followed by 6 months of Standard diet, Agilent Sure Print G3 Mouse Gene Expression 8x60K Arrays in Giller et al. (2013); C57BL/6J mice, 30E% CR with micronutrients supplementation from 2 till 24 months, followed by 4 months of MF diet, Affymetrix GeneChip Mouse Gene 1.1 ST arrays in this study).

One of the most intriguing findings in the current study emerged when we investigated to what extent the CR‐associated genes are linked to their related phenotypes. Previous studies on *Fut8* hepatic expression and plasma glycosylation profile show the modulation of *Fut8* expression and NGA2F, NA2, and NA2F plasma levels during aging and chronic liver diseases (Blomme et al., 2011; Vanhooren et al., 2011). Expression of this gene did not alter during the 4‐month exposure to MF diet, but a slight shift in the fucosylated N‐glycan levels, NGA2F and NA2F, in the plasma was observed. We found that other genes (*Rpn1*,* Rpn2*,* Mogs*,* Hexb*,* Uggt1*), which are all involved in other key steps in N‐glycan biosynthesis/processing, might be responsible for the altered glycosylation profile in the CR‐MF diet switch group. As hepatic steatosis has been reported to disturb the function of ER in hepatocytes (Baiceanu, Mesdom, Lagouge, & Foufelle, 2016), the fact that the mice exposed to CR‐MF diet switch developed hepatic steatosis might contribute to ER stress and stress responses which consequently affected N‐glycan processing in the ER. Another possibility is the presence of other fucosyltransferases, such as *Fut2* and *Fut3* in the gut (Drake et al., 2010), which leads to plasma glycosylation profile modification by multiple tissues.

Another intriguing discrepancy in gene expression and physiological outcome is the hepatic expression of *Cd36*, a fatty acid transport gene which overexpression increases susceptibility to accumulate liver fat (Koonen et al., 2007; Sheedfar et al., 2014). The deletion of this gene has previously been shown to cause resistance to diet‐induced hepatic steatosis (Clugston et al., 2014). In our study, we found that, despite of the development of hepatic steatosis, the expression of *Cd36* in the CR‐MF group remains low, comparable to the expression levels in the lifelong CR diet group. The role of fatty acid transport was seemingly compensated by increased expression of *Cav1* and *Fabp4*. This demonstrates that, after lifelong exposure to a CR diet, the system is still plastic and can adapt to a MF diet, but that alternative genes might be used when a certain function needs to be carried out.

Furthermore, we found that the methylation status of the far upstream enhancer (~40 kbp from the transcription start site), but not of the promoter region of the *Cd36* was affected by the MF diet. Although this observation implies that changes in DNA methylation might be responsible for the altered expression levels of the *Cd36,* our results remain inconclusive. First of all, the methylation levels of the CR‐MF diet switch group are in between those of the CR and MF groups, while the expression levels remain similar to those of the lifelong CR‐exposed mice. Secondly, in the MF intervention group, both gene expression and methylation levels of the enhancer region of *Cd36* were significantly increased, compared to the CR‐fed mice. Decreased *CD36* expression together with decreased DNA methylation has recently been shown in response to a hypocaloric diet‐induced weight loss in female subjects (Do Amaral, Milagro, Curi, & Martínez, 2014). Similar to what we observed, this study showed that gene expression and DNA methylation levels are positively associated. However, the results presented in the study show differential methylation region close to the transcription start site, while in our study, methylation of the enhancer was altered. Further studies are required to unravel the relation between gene expression and DNA methylation for the *Cd36* gene.

To conclude, despite a long term of adulthood CR (22 months), the CR‐MF mice did not become metabolically or physiologically implacably fixed in the state of functioning that CR engendered. The CR‐MF diet switch group developed hyperphagia causing weight gain and hepatic steatosis. Furthermore, the liver transcriptomic profile of CR‐MF group largely shifted to a profile similar to the MF‐fed animals, leaving only ~22% of the 1,578 differentially regulated genes between the CR and MF diet groups comparable with the expression of the lifelong CR group. As illustrated by the analysis of *Cav1*,* Fabp4,* and *Cd36* expressions and their relations with hepatic steatosis, the liver has a robust metabolic network that includes multiple regulators contributing its plasticity in coping with the challenge of MF diet. Therefore, although the diet switch was performed at an old age, the CR‐MF‐exposed mice showed plasticity in adapting to the MF diet without developing severe liver pathologies, which likely contributes to the maintenance of the CR‐improved survival.

## 4 MATERIAL AND METHODS

### 4.1 Ethics statement

The institutional and national guidelines for the care and use of animals were followed and the Local Committee for Care and Use of Laboratory Animals at Wageningen University approved the experiment (code number: drs‐2010151b).

### 4.2 Animals and diets

The animal study was a part of a mice aging cohort (Rusli et al., 2015, 2016). Male C57BL/6J mice (Janvier, Cedex, France) arrived at 7 weeks of age and allowed to acclimate for 2 weeks, receiving standard AIN‐93G (Research Diet Services, Wijk bij Duurstede, the Netherlands) upon arrival. At the start of the diet intervention, the mice were 9 weeks old and randomly distributed into three intervention groups: (i) Control diet receiving AIN‐93W diet *ad libitum* (*n* = 89); (ii) CR diet receiving AIN‐93W‐CR in portions containing 70E% of the mean energy intake of the group of the control mice were provided each day at 15.30 (*n* = 117); (iii) medium‐fat diet (MF) receiving AIN‐93W‐MF *ad libitum* (*n* = 127). AIN‐93W‐CR contained increased concentration of vitamins and minerals content to feed these mice the same concentrations of micronutrients as the mice receiving AIN‐93W diet and avoid malnutrition. Complete diet composition is listed in Table S3 (Research Diet Services, Wijk bij Duurstede, the Netherlands). All mice were provided with *ad libitum* access to water. The mice were housed individually in the light and temperature (20°C)‐controlled animal facility of Wageningen University (12‐h light/dark cycle, light on at 04.00).

The long‐term dietary invention was continued until a sacrifice at the age of 28 month, but in addition to the three diet groups, at 24 month, the animals in the CR diet group were randomly assigned either to remain on the CR diet (*n* = 30) or undergo a diet switch to the MF diet (*n* = 32). This resulted in a group of 25–32 animals in each group at 24 months. Anticipating that the animals would not be used to *ad libitum* feeding after exposure to CR for a long term, the food intake was increased gradually, by addition of 10E% per week. Therefore, it took 3 weeks for the animals to be allowed to have MF *ad libitum*. Body weight of all mice was recorded weekly. Food intake of 20 mice of each intervention group was measured every 2 months. Portion sizes of the mice on the CR were based on the mean food intake of the C‐exposed mice measured for 7 days.

At the age of 28 months, 8–11 mice of each intervention group were sacrificed between 14.00 and 17.00 on three consecutive weeks. Prior to sacrifice, each mouse was first fasted for 4 hr after which they received an intragastric gavage of either Wy‐14,643 (Wy) substance solved in 0.5% carboxymethyl cellulose (CMC) or just the 0.5% CMC solvent and then fasted again for another 6 hr. All mice were used for the phenotypic measurements (bodyweight, food intake, etc.). The aim of the Wy‐treatment was to perform a PPARα adaptive capacity analysis, which has been covered in a separate publication (Rusli et al., 2016). As Wy‐treatment causes an effect on gene expression level, these animals were not included in the transcriptomic analysis. After sedation with isoflurane (1.5%), in a mixture of nitrous oxide (70%) and oxygen (30%), blood samples were collected by cardiac puncture, then followed by neck dislocation. Weight of various organs was measured and snap‐frozen and stored at −80°C until further molecular/biochemical analysis.

### 4.3 Plasma and liver markers analyses

Plasma IL‐6 and CCL2 levels were measured using a Mouse Adipokine (MADKMAG‐71K) kit, while plasma IFNγ, TNF, IL‐1α, IL‐1β, IL‐2, IL‐7, IL‐10, IL‐15, CCL3, CCL4, CCL5, CXCL1, CXCL9 and CXCL10 from Mouse Cytokine (MCYTMAG‐13K) kit (Millipore, Billerica, MA, USA), according to the manufacturer's instructions. Plasma glucose was measured with Glucose GOD FS (DiaSys, Holzheim, Germany), according to the manufacturer's protocol. The insulin and ALT determination in the plasma, as well as the hepatic triglycerides and hydroxyproline analysis, have been previously described (Rusli et al., 2015, 2016).

### 4.4 Microarray hybridization

Prior to the microarray hybridization, total RNA was isolated and checked for its quality (see Appendix S1 for details). Hybridization, washing, and scanning of Affymetrix GeneChip Mouse Gene 1.1 ST arrays were performed according to standard Affymetrix protocols. Microarray analysis was performed in MADMAX, a pipeline for statistical analysis of microarray data (Lin et al., 2011). Arrays were normalized using the Robust Multiarray Average method (Bolstad, Irizarry, Åstrand, & Speed, 2003; Irizarry et al., 2003). Probe sets were defined according to Dai et al. (2005). In this method, probes are assigned to unique gene identifiers, in this case Entrez IDs. The probes on the Gene 1.1 ST arrays represent 21,225 Entrez IDs. For the analysis, only genes having 1) an interquartile range of entire dataset (including all of the groups) of >0.1 and 2) an intensity value of >20 on at least five arrays were taken into account and subsequently investigated for statistical analyses on differences between groups, which resulted in 15,417 genes in the dataset. Array data have been submitted to the Gene Expression Omnibus, with accession number GSE102593.

For the microarray data analysis, differentially expressed probe sets were identified using linear models (library limma) and the intensity‐based moderated *t*‐statistic (IBMT) method was applied. Resulting log_2_ intensities and *p*‐values were used for further descriptive bioinformatic analysis of the data. Heatmap and PCA plots were constructed using MultiExperiment Viewer version 4.8.1 (Saeed et al., 2006) and factomineR package in R, respectively. Pathway and upstream regulator analyses were performed in ingenuity pathway analysis (IPA; Ingenuity^®^ Systems).

### 4.5 Plasma glycomics analysis

N‐glycans on the plasma glycoproteins were analyzed using DNA‐sequencer‐Aided, Fluorophore‐Assisted Carbohydrate Electrophoresis (DSA‐FACE) technology. The same DSA‐FACE protocol used in (Borelli et al., 2015; Vanhooren, Laroy, Libert, & Chen, 2008) for the analysis of the human plasma N‐glycome was applied for the mouse plasma. N‐glycosylation was analyzed in 2 μl of total plasma (see Appendix S1 for detailed procedure). Five major glycan peaks, which had the same migration positions of the well‐known human N‐glycan structures (Fig. S8), were measured using a DNA‐sequencer ABI‐PRISM *3730xl* (Applied Biosystem, Foster City, CA, USA).

### 4.6 Bisulfite conversion and DNA methylation analysis

Genomic DNA was isolated from the liver using the classical proteinase K digestion and phenol: chloroform extraction (see Appendix S1 for detailed preparation). Bisulfite conversion and DNA methylation analysis by means of pyrosequencing were adapted from a previous study (Steegenga et al., 2014). For each sample, 1,000 ng of genomic DNA was bisulfite‐treated using the EZ‐96 DNA Methylation^™^ Kit (Zymo Research, Irvine, CA, USA) and eluted in 60 μl of TE. DNA methylation analysis was performed using PyroMark^™^ pyrosequencing technology (Biotage AB, Uppsala, Sweden). Primers were designed using PyroMark software, and the sequences of the primers used and their specific melting temperature are listed in Table S4. The single‐stranded PCR product (35 μl) is isolated and allowed to hybridize with a sequencing primer, and pyrosequencing was performed using the Q24 Pyrosequencing System (Qiagen, Venlo, the Netherlands) (see Appendix S1 for detailed procedure). CpG methylation was analyzed with the provided software.

### 4.7 Statistical analysis

Statistical analysis, except for the microarray gene expression data, was analyzed with GraphPad Prism 5.04 (Graphpad Software, La Jolla, California, USA). Differences between dietary interventions were analyzed using one‐way ANOVA followed by Tukey post‐test analysis. The alteration of DNA methylation levels in a specific region was evaluated with two‐way ANOVA followed by Bonferroni post‐test analysis. Results represented in bar graphs are shown as means ± standard deviation. For results plotted in box‐and‐whisker plots, the box extends from 25^th^ to 75^th^ percentiles with a line at median value, while the whiskers denote 5 and 95 percentiles. Pearson's correlation was used to determine the relationship between variables. Statistical significance for the survival of groups was established by the log‐rank analysis of Kaplan–Meier plots.

## CONFLICT OF INTEREST

None declared.

## AUTHOR CONTRIBUTIONS

FR, MM, and WTS conceived and designed the experiments. FR, CL, MVB, VB, CS, ALM, JvdH, and WTS performed the experiments and data analyses. FR and WTS wrote the manuscript. MVB, ALM, JvdH, SS, CF, and MM provided valuable feedback on the manuscript.
