# Supplementary material for: Plasticity of lifelong calorie‐restricted C57BL/6J mice in adapting to a medium‐fat diet intervention at old age
Source: Aging Cell. 2017 Dec 21;17(2):e12696. doi: 10.1111/acel.12696 (PMC5847878; doi:10.1111/acel.12696)
Supplement: Supplementary file 1 [file ACEL-17-e12696-s001.pdf]

Table S1. 10 most significantly different canonical pathways differentially regulated/expressed between the life-long CR and MF intervention groups

Top 10 canonical pathways CR vs MF	*p*-value
Mitochondrial dysfunction	9.56×10^{-9}
Oxidative phosphorylation	1.04×10^{-8}
Apoptosis signaling	3.25×10^{-7}
Production of NO and ROS in macrophages	2.75×10^{-5}
IL-4 signalling	3.12×10^{-5}
MSP-RON signalling pathway	3.72×10^{-5}
Rac signalling	5.82×10^{-5}
TNFR1 signalling	7.31×10^{-5}
Sphingosine-1-phosphate signaling	1.12×10^{-4}
Tumoricidal function of hepatic natural killer cells	1.14×10^{-4}

Table S2. Gene expression difference for biosynthesis N-glycan genes and their correlation to the N-glycan proteins

	Significance[a]			Correlation[b]		
**Gene name**	CR vs MF	CR-MF vs CR	CR-MF vs MF	NGA2F	NA2	NA2F
**N-glycan quality control**						
Canx	ns	*	*	-0.019	-0.349	-0.208
Calr	ns	ns	ns	-0.222	-0.123	-0.510*
Hspa5	ns	ns	ns	0.451	-0.521*	-0.020
Pdia3	*	ns	ns	-0.346	0.022	-0.536*
Hsp90b1	*	ns	ns	-0.154	-0.123	-0.390
Uggt1	**	*	ns	-0.276	0.003	-0.583**
**Glycan molecules synthesis genes**						
Dpagt1	**	***	ns	-0.031	0.265	-0.272
Alg13	ns	ns	ns	-0.008	0.047	-0.073
Alg14	ns	ns	ns	-0.365	0.084	-0.082
Alg1	ns	**	ns	0.059	0.201	-0.202
Alg2	ns	ns	ns	0.353	-0.093	-0.123
Alg12	*	ns	ns	0.037	-0.127	0.133
Alg3	ns	**	*	-0.246	0.384	-0.096
Alg11	**	ns	ns	-0.492*	0.336	-0.253
Alg9	ns	ns	*	0.165	-0.173	0.087
Alg6	ns	ns	ns	-0.038	0.268	-0.067
Alg10b	ns	ns	ns	0.226	-0.047	0.120
**Glycans-to-asparagine residues transfer**						
Ddost	ns	***	**	-0.113	-0.089	-0.150
Rpn1	*	ns	*	-0.541*	0.234	-0.493*
Rpn2	**	**	ns	-0.252	0.141	-0.584**
Dad1	ns	*	ns	-0.241	0.204	-0.377
Tusc3	ns	ns	ns	0.009	0.283	0.109
**Trimming of the N-glycan in the ER and *cis*-golgi**						
Man2a1	ns	ns	ns	-0.004	-0.031	-0.296
Man2a2	ns	ns	ns	0.148	0.276	-0.055
Man1b1	ns	ns	ns	0.099	-0.223	-0.317
Man1a2	ns	ns	ns	-0.195	-0.193	0.036
Man1c1	*	ns	ns	0.244	-0.263	0.054
Man1a	ns	ns	ns	0.130	-0.178	0.195
Ganab	ns	ns	*	-0.216	-0.122	-0.441
Mogs	*	*	ns	-0.131	0.577**	-0.143
**Glycan extension**						
Fut8	**	ns	***	0.183	-0.417	0.293
Mgat1	ns	*	ns	-0.368	0.303	-0.485*
Mgat4a	*	ns	ns	0.355	-0.385	0.287
Mgat5	*	*	ns	0.243	-0.485*	-0.076
Mgat2	ns	ns	ns	0.218	-0.094	-0.072
B4galt1	ns	ns	ns	-0.185	-0.266	-0.175
B4galt2	ns	ns	ns	0.024	-0.124	0.185
B4galt3	ns	*	ns	-0.357	0.353	-0.343
St6gal1	**	ns	ns	0.165	-0.455	-0.099
St8sia2	ns	ns	ns	-0.233	0.065	-0.244
St8sia3	***	ns	*	0.096	-0.496*	0.004
St8sia4	**	ns	ns	0.488*	-0.428	0.444
**N-glycan degradation**						
Aga	ns	ns	ns	0.015	-0.039	0.259
Fuca1	ns	ns	ns	-0.097	0.191	0.437
Fuca2	**	**	ns	0.218	-0.235	0.065
Engase	ns	ns	ns	-0.087	0.118	-0.259
Manba	ns	ns	ns	-0.210	0.134	-0.026
Man2b1	ns	ns	ns	0.060	0.129	-0.144

Table S2 (continued). Gene expression difference for biosynthesis N-glycan genes and their correlation to the N-glycan proteins

	Significance[a]			Correlation[b]		
**Gene name**	CR vs MF	CR-MF vs CR	CR-MF vs MF	NGA2F	NA2	NA2F
**N-glycan degradation (continued)**						
Man2b2	***	*	ns	0.212	-0.462*	0.123
Man2c1	*	**	ns	-0.132	0.197	-0.325
Hexa	***	*	ns	0.216	-0.267	0.296
Hexb	***	**	***	0.308	-0.589**	0.313
Glb1	ns	ns	ns	-0.043	0.310	-0.287
Neu1	ns	ns	*	0.283	-0.134	-0.250
Neu2	ns	ns	ns	-0.090	0.296	0.337
Neu3	ns	ns	*	0.292	-0.515*	0.257

[a]) Statistical difference between the diet groups was determined by intensity-based moderated t-statistic (IBMT) *p*-value. *$p < .05$; **$p < .01$; ***$p < .001$.

[b]) r values and their significance were calculated with Pearson's correlation. *$p < .05$; **$p < .01$.

Table S3. Composition of the experimental diet. The CR diet was adjusted for the vitamins and minerals amount to ensure a homologous intake between both groups.

	**AIN-93W**	**AIN-93W-CR**	**AIN-93W-MF**
Energy (kcal/g)	3.85	3.77	4.25
Energy from fat (%)	9	10	25
Energy from protein (%)	15	15	13
Energy from carbohydrates (%)	76	75	61
Mineral mix AIN-93M (g%)	35	50	35
Vitamin mix AIN-93M (g%)	10	14	10
Choline bitartrate (g%)	2.5	3.5	2.5

Table S4. Pyrosequencing primers details of *Cd36* promoter and enhancer region

Gene	Chromosomal position	Primer sequence (5‘ to 3’)	Annealing temperature (°C)	PCR product size (bp)
Cd36 promoter	ch5:17341776-17341855	FP: AAGTTTATAAGGGTTTATTTTTGGTGAAG<br>RP: ACTCCAACATCTAAAATAACAATTACAAT<br>SP: GTTTTTATTTAAGTAAGTTAGAGG	57.8	205
Cd36 enhancer	ch5:17435129-17435227	FP: TTGTTGTGTTAGGGAAATATTAATGAT<br>RP: ATAACTCCAACACCAACCACAATAA<br>SP: AGGGTAGTTATTTTAGTTAAGTTAG	58.6	265

Figure S1

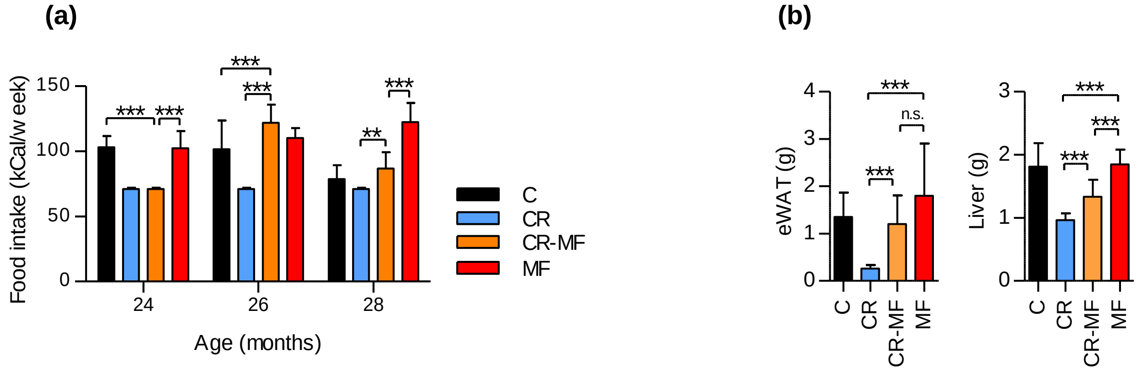

Figure S1. (a) Food intake measurement at 24, 26 and 28 months. Statistical significance was assessed by 1-way ANOVA followed by Tukey post-test analysis for each time point. (b) eWAT and liver weight at 28 months. Statistical significance of CR-MF difference to CR and MF was assessed by 1-way ANOVA followed by Tukey post-test.

Figure S2

(a)

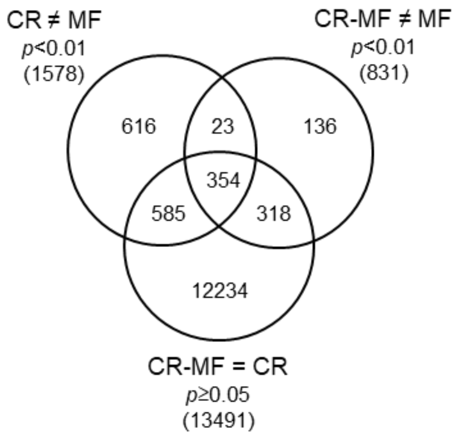

(b)

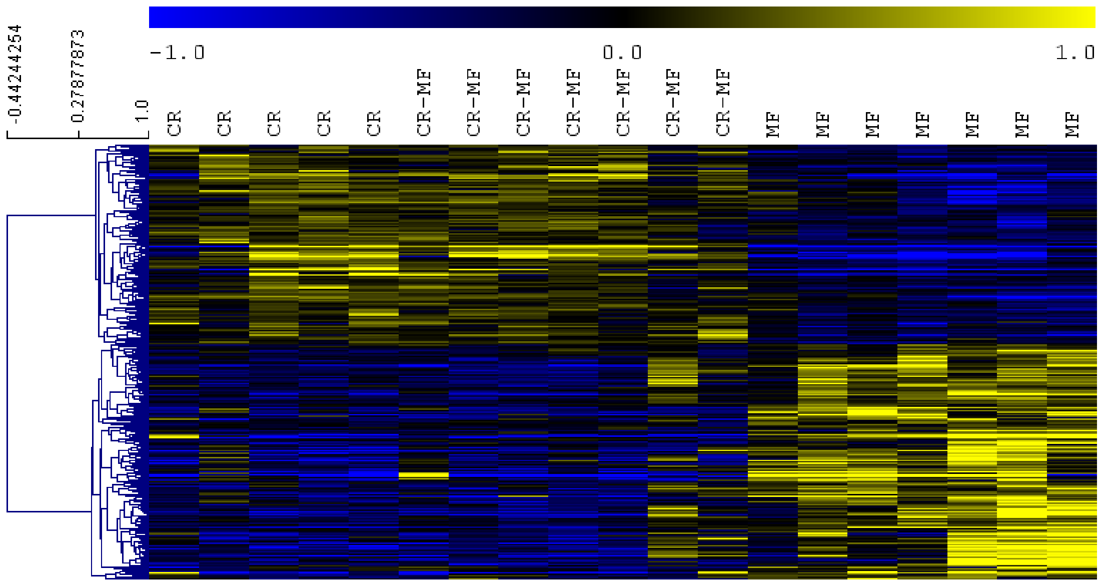

Figure S2. (a) Venn diagram visualizing overlaps of the genes differentially expressed between CR vs MF, CR-MF vs MF and the genes remained similar between CR-MF vs CR. This is another approach to identify the 354 CR-associated genes in CR-MF expression presented in Figure 2b. (b) Heatmap comparing the expression of 354 CR-associated genes in the CR, CR-MF and MF diet groups.

Figure S3

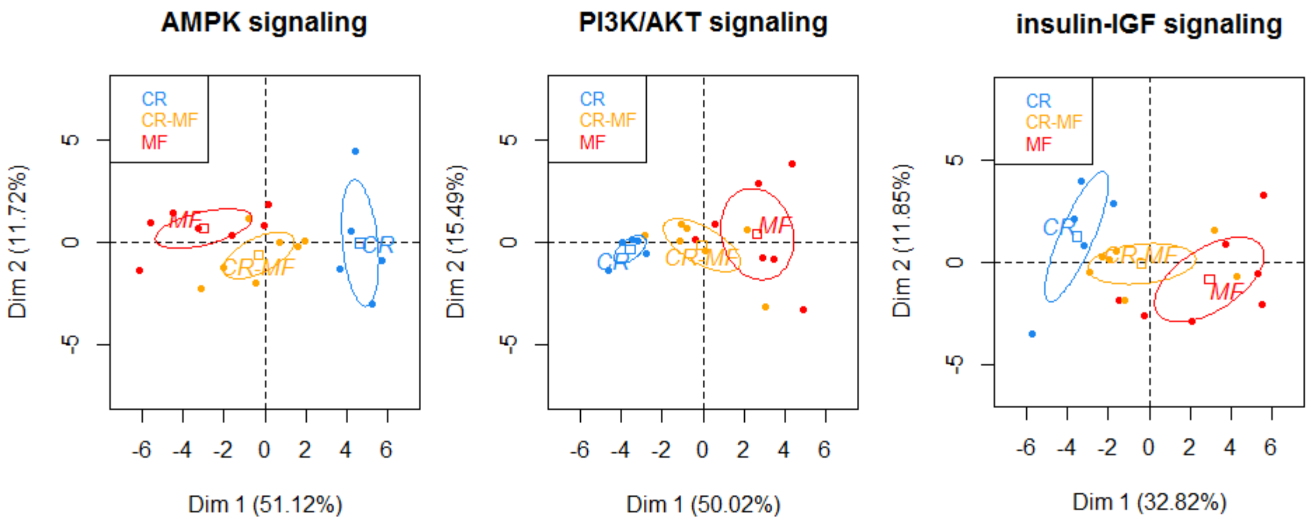

Figure S3. PCA plots of gene sets revealing the shift of the gene expression profile of specific signalling pathways following the CR-MF diet switch: AMPK signalling, PI3K/AKT signalling and insulin-IGF signalling. The expression values of all three dietary interventions were normalized to the C group.

Figure S4

(a)

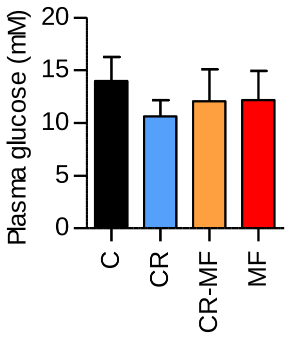

(b)

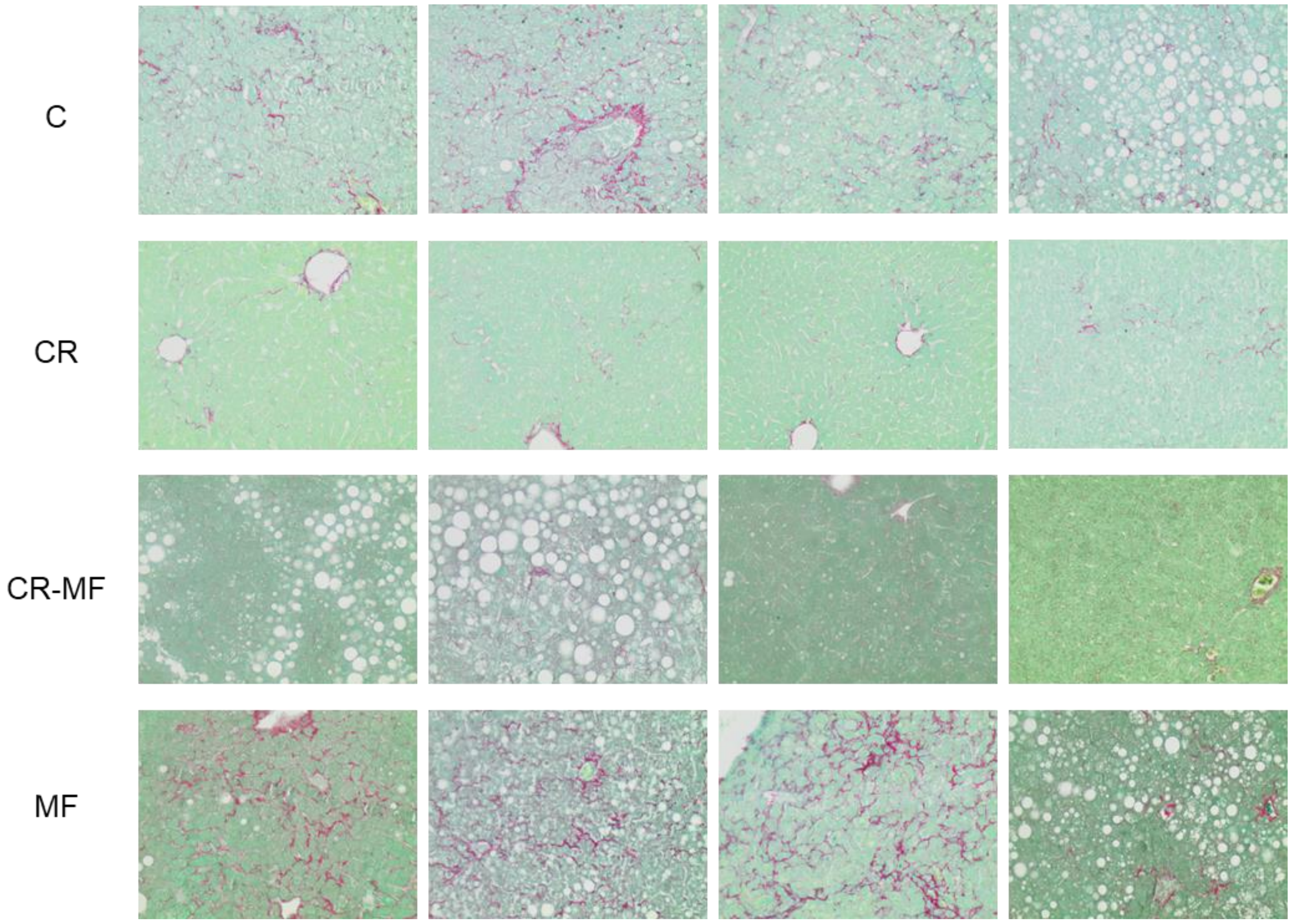

Figure S4. (a) Fasting plasma glucose measurement shows that there was no significant difference between the different diet groups. Statistical analysis was performed using 1-way ANOVA followed by Tukey post-test. (b) Collagen staining on liver sections of mice exposed to C, CR, CR-MF and MF diet, which extends Figure 3E (original magnification 200x). The formalin-fixed and paraffin-embedded sections (5 µm) of the liver lobe was stained with fast green FCF and Sirius Red. Each histology images in this figure and Figure 3e represents different mice.

Figure S5

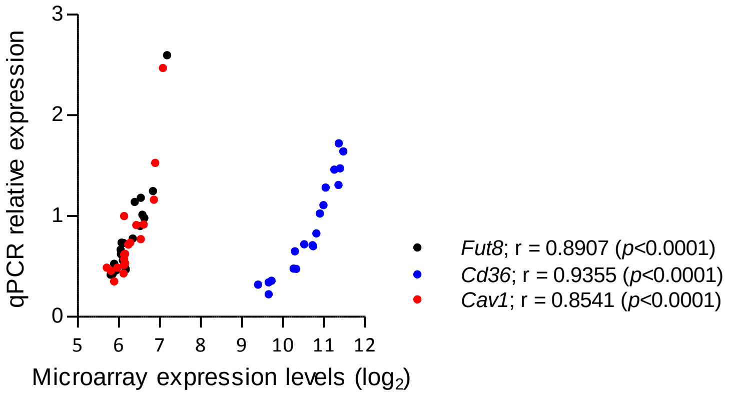

Figure S5. Correlation between microarray and qPCR gene expression of *Fut8*, *Cd36* and *Cav1*. r values and their significance (in parentheses) were calculated with Pearson's correlation.

Figure S6

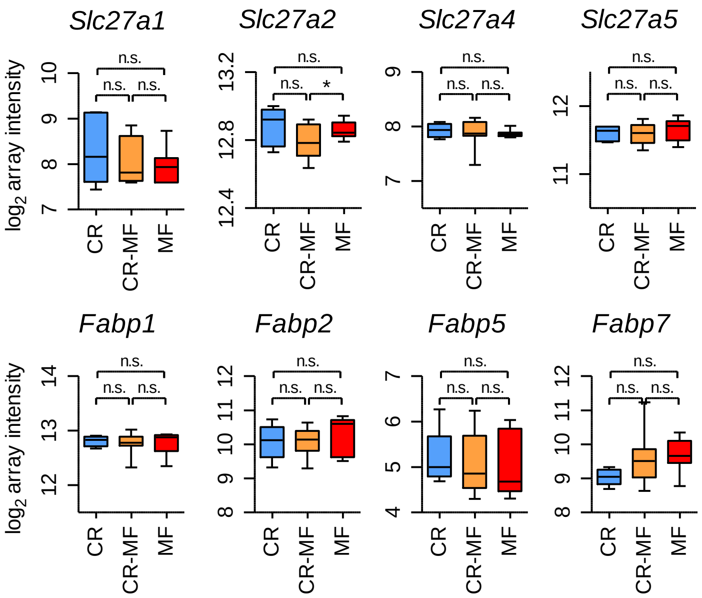

Figure S6. The expression levels of fatty acid transporter/uptake genes in CR, CR-MF and MF diet group. No statistical difference was found for these genes, which was determined by intensity-based moderated t-statistic (IBMT) *p*-value.

Figure S7

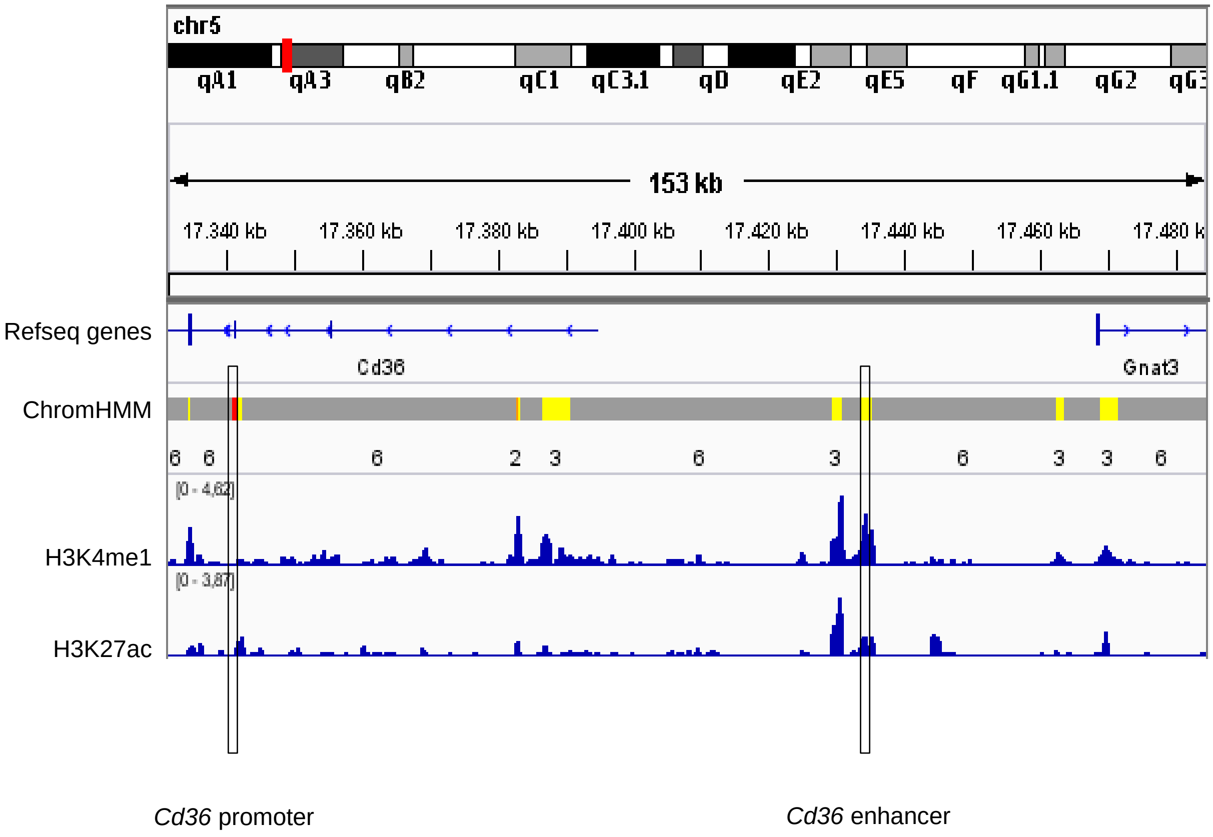

Figure S7. Chromosomal position of regions analysed for differential DNA methylation level within and upstream of *Cd36* gene. Epigenetic features ChromHMM, H3K4me1 and H3K27ac were obtained from the mouse ENCODE database and visualized in integrative genomics viewer (IGV, Broad Institute). The red and yellow colors in ChromHMM track represent promoter and weak enhancer regions, respectively. The regions selected for DNA methylation analysis by pyrosequencing method are marked with boxes.

Figure S8

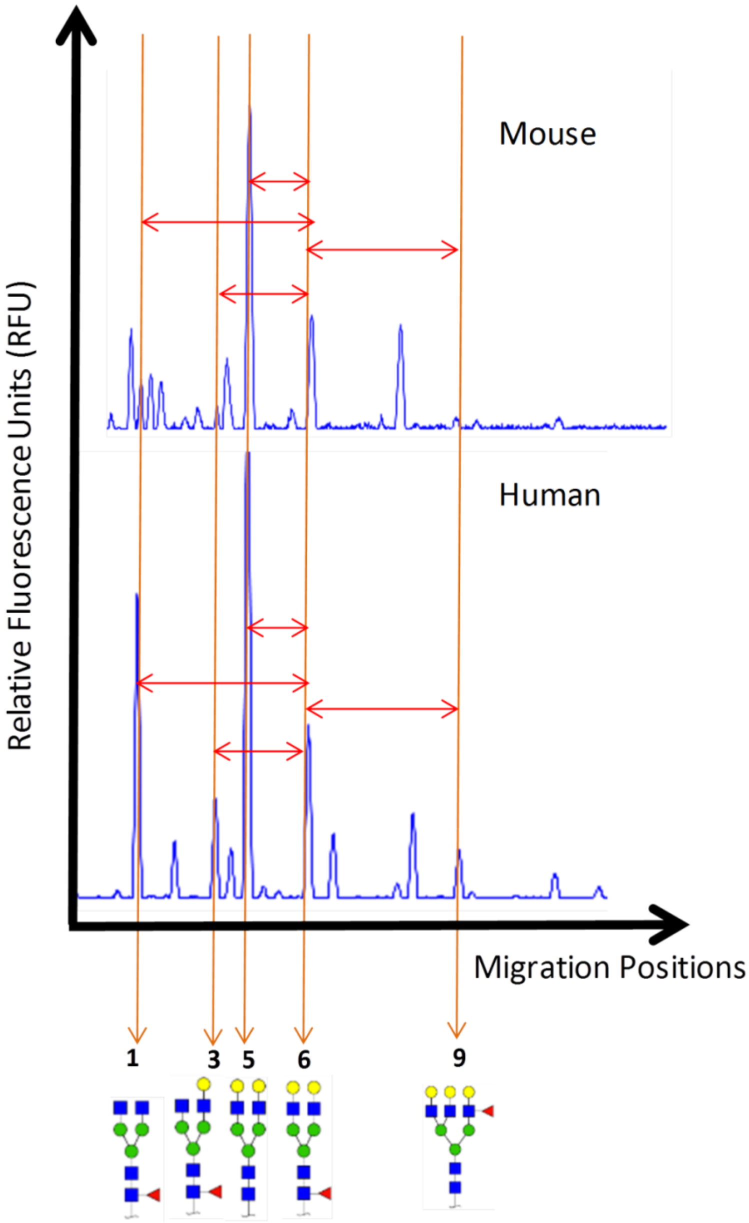

Figure S8. Comparison of glycan migration position in mouse and human samples. Five major glycan peaks had the same migration positions of the well-known human N-glycan structures, i.e. peak 1 (NGA2F, an agalactosylated core-α-1,6-fucosylated, biantennary N-glycan), peak 3 (NG1A2F, a mono-galactosylated core- α-1,6-fucosylated, biantennary N-glycan), peak 5 (NA2, a bigalactosylated, biantennary N-glycan), peak 6 (NA2F, a bigalactosylated, core-α-1,6-fucosylated biantennary N-glycan) and peak 9 (NA3, a triantennary, trigalactosylated N-glycan).

Appendix S1

RNA isolation

Total RNA was isolated using TRIzol reagent (Invitrogen, Breda, The Netherlands), according to manufacturer's instructions. The RNA was treated with DNAse and purified on columns using the RNeasy microkit (Qiagen, Venlo, The Netherlands). RNA concentration was measured on a NanoDrop ND-1000 UV–vis spectrophotometer (Isogen, Maarsen, The Netherlands) and RNA integrity was checked on an Agilent 2100 Bioanalyzer (Agilent Technologies, Amsterdam, The Netherlands) with 6000 Nano Chips, according to manufacturer's instructions. RNA was judged as suitable only if samples showed intact bands of 18S and 28S ribosomal RNA subunits, displayed no chromosomal peaks or RNA degradation products and had a RNA integrity number (RIN) above 8.0.

cDNA synthesis and real-time quantitative PCR

The microarray data was validated by real-time quantitative PCR (qPCR) for genes *Fut8*, *Cd36* and *Cav1*. For each individual sample, single-stranded complementary DNA (cDNA) was synthesized from 1 µg of total RNA using the First Strand cDNA Synthesis kit (Thermo Scientific, Landsmeer, the Netherlands), following the supplier's procedure. qPCR was performed using SensiMix SYBR No-ROX kit (Bioline, Alphen aan de Rijn, the Netherlands) and a CFX384 thermal cycler (Bio-Rad, Veenendaal, the Netherlands). The following thermal cycling conditions were used: 2 min at 94ºC, followed by 40 cycles of 94ºC for 15s and 60ºC for 45s. PCR reactions were performed in duplicate and all samples were normalized to *Rplp0* expression. Primer sequences are listed as below.

**Gene name**	**Forward primer (5' → 3')**	**Reverse primer (5' → 3')**
Fut8	GCTTCTCGCACGCAGAATG	GTGTACCGATTGTGTAGTCCAG
Cd36 antigen	TCCAGCCAATGCCTTTGC	TGGAGATTACTTTTCAGTGCAGAA
Cav1	AACATCTACAAGCCCAACAACAAGG	GGTTCTGCAATCACATCTTCAAAGTC

Plasma glycomics analysis

Total plasma of 2 µl volume was incubated for 5 min at 95°C with 2 µl of 5% SDS in 10 mM NH4HCO3. The N-glycans on the plasma glycoproteins were enzymatically released with 33 Units of PNG-ase F (New England Biolabs,Ipswich, MA, USA) in 3 µl of 3.33% NP-40 and 10 mM NH_4HCO_3, pH 8.3 and incubated for 3 h at 37°C. The released N-glycans were desialylated with 2 mU of Neuraminidase of *Arthrobacter ureafaciens* (Roche Diagnostics GmbH, Mannheim, Germany) in 5mM NH_4Ac, pH 5, incubating for 3 h at 37°C. Subsequently, 2 µl of the desialylated N-glycans in 5 µl of water were dried completely at 60°C for 1 h. After that, 2 µl of a labeling solution (1:1 mixture of 20 mM of fluorophore 8-amino-1,3,6-pyrenetrisulfonic acid (APTS) (Life Technologies, Carlsbad, CA, USA) in 1.2 M citric acid and 1 M $NaCNBH_3$ in DMSO) were added per sample and incubated overnight at 37°C. The reaction was stopped with 150 µl of water to each well. This solution was diluted 1:2.5 in water and 2 µl of this solution were added to 8 µl of D-Formammide for the sequencing analysis in a DNA-sequencer ABI-PRISM *3730xl* (Applied Biosystem, Foster City, CA, USA). The electopherogram of the mouse plasma N-glycans profile contained several peaks that were structurally characterized by overlapping their migration positions with those previously measured in the human plasma profile. Five major glycan peaks had the same migration positions of the well-known human N-glycan structures (**Fig. S8**), in particular, peak 1 (NGA2F, an agalactosylated core- α -1,6-fucosylated, biantennary N-glycan), peak 3 (NG1A2F, a mono-galactosylated core- α -1,6-fucosylated, biantennary N-glycan), peak 5 (NA2, a bigalactosylated, biantennary N-glycan), peak 6 (NA2F, a bigalactosylated, core-α-1,6-fucosylated biantennary N-glycan) and peak 9 (NA3, a triantennary, trigalactosylated N-glycan). The heights of these peaks, that represent the relative concentrations of the N-glycan structures, were quantified and normalized to the total signal intensity by using Peak Scanner software (Applied Biosystem,Foster City, CA, USA).

DNA isolation from the liver tissue

Genomic DNA was isolated from the liver by using the classical proteinase K digestion and phenol:chloroform extraction. The DNA was treated with RNase and eluted in RNase and DNase free distilled water. DNA purity and quantity were checked spectrophotometrically with NanoDrop ND-1000 (NanoDrop Technologies, Wilmington, USA) and fluorometrically with Qubit DNA (Invitrogen, Oregon, USA).

Bisulfite conversion and DNA methylation analysis

Genomic DNA was isolated from the liver by using the classical proteinase K digestion and phenol:chloroform extraction (see Supplementary Materials and Methods for detailed preparation). Bisulfite conversion and DNA methylation analysis by means of pyrosequencing were adapted from a previous study (Steegenga *et al.* 2014). For each sample, 1000 ng of genomic DNA was bisulfite-treated using the EZ-96 DNA Methylation™ Kit (Zymo Research, Irvine, CA, USA) and eluted in 60 µl of TE. DNA methylation analysis was performed using PyroMark™ pyrosequencing technology (Biotage AB, Uppsala, Sweden). Primers were designed using PyroMark software, and the sequences of the primers used are listed in Table S4. The PCR reactions were performed in a total volume of 45 µl, and the volume of bisulfite-treated genomic DNA used was 4 µl. PyroMark PCR Master Mix and CoralLoad Concentrate were used according to the manufacturer's instructions, and 0.2 µM of each primer (Qiagen, Venlo, The Netherlands) was used. The following thermal cycling conditions were applied: 15 min at 95°C, followed by 45 cycles of 94°C for 30 s, tempX (gene-specific, see Table S4) for 30 s, and 72°C for 40 s, followed by a final elongation step at 72°C for 10 min. The PCR product (35 µl) was bound to Streptavidin Sepharose HP beads (GE Healthcare, Uppsala, Sweden) and purified and made single-stranded using the Pyrosequencing Vacuum Prep Tool according to the manufacturer's instructions (Qiagen, Venlo, The Netherlands). Sequencing primers (Table S4) were annealed to the purified single-stranded PCR product, and pyrosequencing was performed using the Q24 Pyrosequencing System (Qiagen, Venlo, The Netherlands). CpG methylation was analyzed with the provided software.
